# A novel enterovirus in lambs with poliomyelitis and brain stem encephalitis

**DOI:** 10.1111/tbed.14412

**Published:** 2021-12-21

**Authors:** Herbert Weissenböck, Arnt Ebinger, Anna Maria Gager, Denise Thaller, Dirk Höper, Katharina Lichtmannsperger, Christiane Weissenbacher‐Lang, Julia Matt, Martin Beer

**Affiliations:** ^1^ Institute of Pathology Department of Pathobiology Vienna Austria; ^2^ Friedrich‐Loeffler‐Institut Federal Research Institute for Animal Health Institute of Diagnostic Virology Greifswald Germany; ^3^ University Clinic for Ruminants Vienna Austria

**Keywords:** encephalomyelitis, enterovirus, in situ hybridization, lamb, next‐generation sequencing

## Abstract

An Austrian organic dairy sheep farm experienced cases of recumbency and sudden deaths in 3‐ to 4‐week‐old lambs. Two animals were subjected to thorough clinical and pathological investigations. Pathohistological analysis identified severe nonsuppurative myelitis and mild nonsuppurative encephalitis. A reverse‐transcription quantitative PCR (RT‐qPCR) assay for the recently discovered ovine picornavirus causing comparable lesions scored negative. By next‐generation sequencing‐based metagenomics, a nearly complete genome of a novel enterovirus could be detected and assembled. In situ hybridization using a specifically designed probe revealed robust signals in affected motoneurons of the spinal cord suggesting a causative role of the novel virus.

## 1 INTRODUCTION

Encephalomyelitis in sheep has been associated with several viral and few bacterial pathogens. The most common viral agents are small ruminant retroviruses (Maedi/visna virus, caprine arthritis encephalitis virus), several tick‐ and mosquito‐borne flaviviruses (tick‐borne encephalitis virus, louping ill virus, Spanish sheep encephalitis virus, Spanish goat encephalitis virus, West Nile virus), Borna disease virus, and ovine astroviruses (Böhm et al., 2017; Caplazi & Ehrensperger, 1998; Rimoldi et al., 2017; Salinas et al., 2017; Wildi & Seuberlich, 2021; Wolf, 2021). Among the bacterial agents, *Listeria monocytogenes* is most frequently found, and other bacteria such as *Escherichia coli* or *Citrobacter freundii* have been identified in cases of ovine (meningo)encephalitis (Liu et al., 2018; Oevermann et al., 2010; W. Wang et al., 2020). Recently, Forth et al. (2019) described a series of cases of recumbent preweaned lambs that had severe nonsuppurative encephalomyelitis with neuronal replication of a novel picornavirus. The authors suggested that this infection maybe widespread, but usually subclinical and self‐limiting, particularly in older animals. The fact that the disease was only recognized in artificially reared lambs, which did not receive colostrum, indicated likely failure of colostral antibody transfer. It has been hypothesized that clinical disease due to this virus is rare because of herd immunity, which is not transferred to lambs deprived of colostral uptake. These cases had been found exclusively in the United Kingdom so far. In the present contribution, we describe an episode of ataxia progressing into paralysis and recumbency in a herd of lambs in Austria, with pathological lesions indistinguishable from the ones reported earlier.

## 2 MATERIALS AND METHODS

### 2.1 Herd history

The cases occurred in an organic dairy sheep farm located in the northern area of Lower Austria. The Lacaune dairy sheep herd is free of numerous viral and bacterial infectious diseases including Maedi Visna, Border disease, *Brucella*, and *Corynebacterium pseudotuberculosis*. Since 2019, the ewes are vaccinated against *Erysipelothrix rhusiopathiae* since cases of red murrain have been diagnosed in the herd (Schoiswohl et al., 2020). The 130 dairy sheep are housed in a free stall, which was built in 2018. All ewes give birth to the lambs in late autumn. In case newborn lambs show a well‐developed mother‐lamb relationship (physiological general behaviour, adequate colostrum/milk intake), they are allowed to stay with their ewes for 5–10 days following birth. Otherwise, they are artificially reared which means that they are separated and fed from an artificial milk provider. Female lambs are kept as young stock at the farm and the majority of males are sold for fattening to another farm at the age of 10 days or stay at the farm to be sold with approximately 40 kg.

### 2.2 Case presentation

**FIGURE 1:**
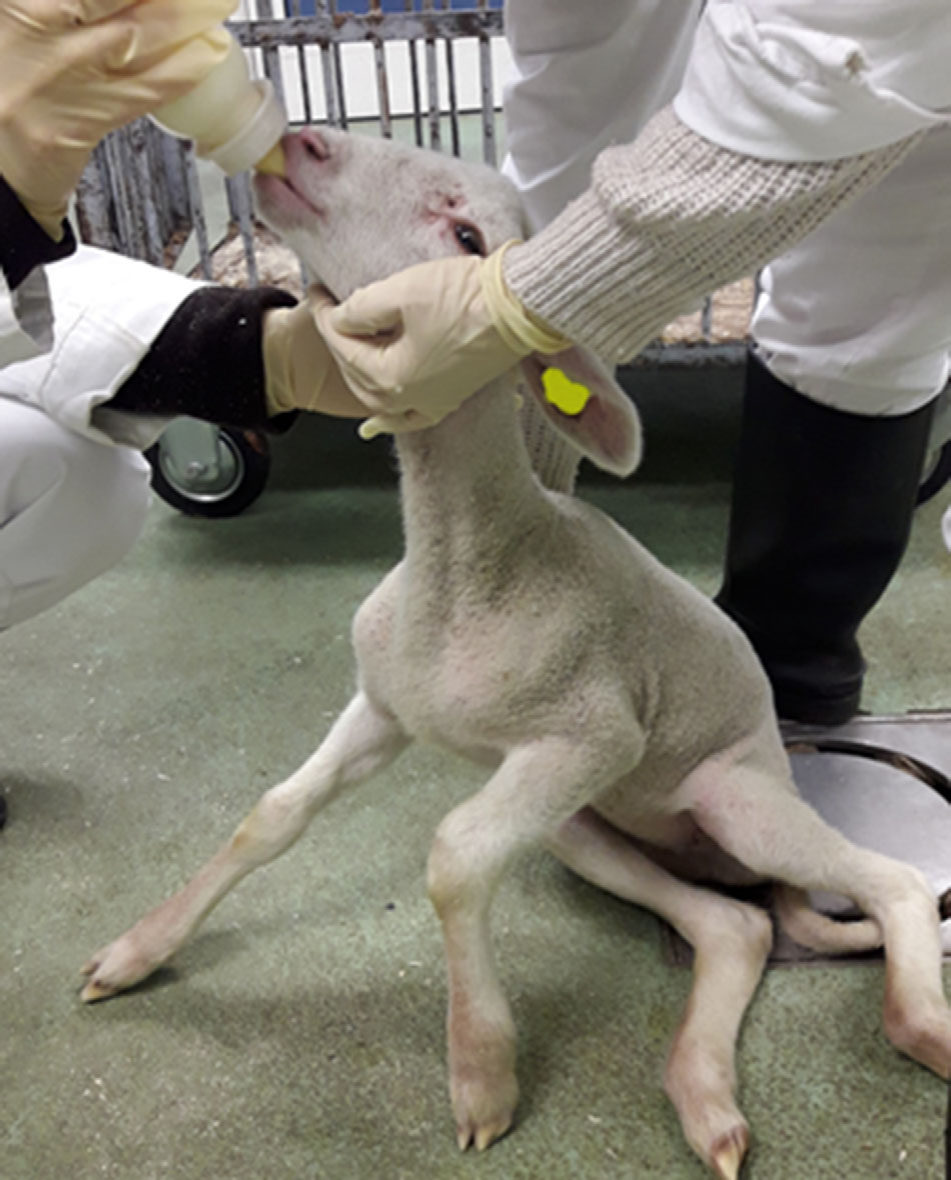
Lamb 1 showing hind limb paralysis and physiological milk intake

On 5 November 2019, the farmer consulted the University Clinic for Ruminants, Vienna since he recognized sudden deaths in male and female lambs aged between 3 to 4 weeks, all born between 12 October and 22 October. The sudden deaths were observed solely at the farm of origin, not at the fatteners. Four lambs died within 24 h, initially showing elevated body temperature followed by decreased body temperature and progressive neurological symptoms. The first signs were hind limb weakness, followed by paralysis of the hind limbs, with preservation of milk and feed ingestion (Figure 1). Later, the animals became recumbent, showed torticollis and/or opisthotonus and died within 24 h. One female (lamb 1; 17 days; 8 kg) and one male (lamb 2; 24 days; 9 kg) Lacaune lamb were presented at the University Clinic for Ruminants for herd diagnosis. One of the animals was artificially reared due to postnatal weakness and questionable colostrum intake. The medical history was not clear enough to verify which of the animals had a lack of colostrum intake. Lamb 1 showed hind limb paralysis (Figure 1), no deep sensibility and muscular tone, and an increased body temperature (40.1°C). Lamb 2 was recumbent and nibbling at the ground (body temperature: 35.0°C). Ethylenediamine tetraacetic acid (EDTA)‐treated and serum blood samples were collected for differential blood count and minerals status. Results showed a decreased packed cell volume of 22.6% (reference: 28.0%−39.0%) and 1122/μl lymphocytes (reference: 2400/μl −9000/μl). Copper and selenium values were within the physiological range (copper: 9.0–25.0 μmol/L; selenium: 53.0–176.0 μmol/L) in both animals. Both lambs were immediately euthanized using pentobarbital (90 mg/kg, Release 300 mg/ml, Wirtschaftsgenossenschaft Deutscher Tierärzte eG) and were referred to the Institute of Pathology for further diagnosis.

### 2.3 Necropsy and histological examination

Both animals were necropsied according to standard protocols. A set of tissue samples including brain and spinal cord were fixed in 10% neutral buffered formalin, embedded in paraffin wax, sectioned at 2–3 μm, and stained with haematoxylin and eosin.

### 2.4 Next‐generation sequencing and sequence analyses

Frozen brain and spinal cord samples of lamb 1 were used for next‐generation sequencing (NGS)‐based metagenomics. Sample preparation was performed as described previously (Wylezich et al., 2018) with a few changes. Briefly, after disintegration of the brain tissue by cryoPREP (Covaris) treatment, RNA was extracted with an RNAdvance Tissue Kit (Beckman Coulter) according to the manufacturer's instructions on a KingFisher Flex (Thermo Fisher Scientific) instrument. The extracted RNA was quantified with a Nanodrop ND1000 (PeqLab), and 500 ng RNA was used as input for cDNA and second strand synthesis using a SuperScript IV First‐Strand Synthesis System (Life Technologies) and the NEBNext Ultra II Non‐Directional RNA Second Strand Synthesis Module (NEB). Thereafter, the resulting double‐stranded cDNA was fragmented with a Covaris M220 focused sonicator (Covaris), and the fragmented DNA was used as input for library preparation, which was performed with a GeneRead DNA Library L Core Kit (Qiagen) and an Ion Xpress Barcode Adapter (Life Technologies). The generated Ion Torrent compatible libraries were quantified with the KAPA Library Quantification Kit ‐ Ion Torrent Universal (Roche) and then sequenced with an Ion Torrent S5 XL (Thermo Fisher Scientific) on an Ion 530 chip in 400 Bp mode according to the manufacturer's instructions. For the initial taxonomic binning, the resulting dataset was analyzed using RIEMS (Scheuch et al., 2015). Since the virus content of the library was low, additional sequencing (Ion Torrent S5 XL, Ion 540 Chips) was performed as per the manufacturer's instructions in order to generate enough data for the assembly of a complete viral genome. The genome sequence was assembled from the combined datasets applying an iterative hybrid approach. First, reads representing the enteroviral genome were identified using Diamond blastx (Buchfink et al., 2015) and all to date available related picornaviral sequences. These reads were assembled de novo using Newbler (v 3.0; Roche). In subsequent mappings (Newbler, v3.0; Roche), additional reads were identified and the genome sequence was re‐assembled until the nearly complete genome sequence was obtained. The sequence is available from the INSDC databases under study accession PRJEB47260.

### 2.5 Phylogenetic analysis

For phylogenetic analysis, a representative panel of complete genome sequences of related species (GenBank accession numbers LC316827, KT265911, KY761948, AF363455, AF363453, JQ818253, JN807387, HQ702854, MN598038, MG958646, KU297674, JQ277724, KU172420, DQ092794, AY843306) or polyprotein amino acid sequences derived thereof were aligned using MAFFT (Katoh & Standley, 2013; Katoh et al., 2002). Phylogenetic reconstruction was done with IQ‐TREE multicore version 1.6.12 (Nguyen et al., 2015) applying automatic best model selection using ModelFinder (Kalyaanamoorthy et al., 2017) included in IQ‐TREE and 100.000 ultrafast bootstraps (Hoang et al., 2018).

### 2.6 Reverse‐transcription quantitative PCR

This method was used for investigating the tissue distribution of the virus in formalin‐fixed and paraffin wax‐embedded tissue samples. From each block, five sections with a thickness of 10 μm were placed in a 1.5 ml tube. RNA was extracted from FFPE material using the truXTRAC FFPE total NA Kit (Covaris) in combination with the Agencourt RNAdvance Tissue Kit (Beckman Coulter) and a KingFisher Flex instrument (Thermo Fisher Scientific). From the initial draft sequence, a reverse‐transcription quantitative PCR (RT‐qPCR) assay was designed. As a positive control, a gBlock oligo (Integrated DNA Technologies) was used. As RT‐qPCR was performed with a SensiFAST Probe No‐ROX Kit (Meridian) according to the manufacturer's instructions. The PCR was run as a duplex assay containing an internal control assay as described (Hoffmann et al., 2006) and for the detection of the novel enterovirus primers EV‐f5 (5′‐ AAACGTTGATTTCGCCCGTG‐3′) and EV‐r1 (5′‐ GTGGATAAATCAGGGCGTTG‐3′), final concentration 0.4 μM each, and probe EV‐p1 (5′‐ 6FAM‐TTCAGTGCCCGCCTTCCCCG‐BHQ‐1‐3′), final concentration 0.2 μM. The oligonucleotides were designed and checked for specificity with primer‐blast algorithm (Ye et al., 2012). As a positive control, a gBlock oligo (Integrated DNA Technologies) was used. For this, the amplicon framed by the primers was inserted into the double‐stranded DNA synthetic gBlock oligo. The concentration of the positive control was set to 1000 c/μl, corresponding to a Cq value of 29.22 when testing different samples from the two affected animals. The reaction was performed using a Bio‐Rad CFX96 real‐time cycler (Bio‐Rad) with the following cycling conditions: (1) Reverse Transcription, 10 min at 45°C; (2) DNA denaturation and activation of the polymerase for 2 min at 95°C; and (3) 45 cycles of 5 s at 95°C (denaturation) followed by 20 s at 60°C (elongation; data acquisition).

### 2.7 In situ hybridization

Chromogenic in situ hybridization (ISH) was performed according to a previously published protocol (Stadler et al., 2015) using a final probe concentration of 20 ng/ml. The oligonucleotide probe was designed based on the nearly complete genome sequence, located within the 5´‐UTR (bases 196–240; sequence 5′‐ACTGAGCCGCTATTGGTCGATTGGTGTGCGTGCTTCTAAGTTACG −3′). Unintentional cross‐reactivity with other organisms was excluded in silico by NCBI BLAST and in vitro using an irrelevant probe targeting the nucleocapsid gene of porcine epidemic diarrhoea virus.

## 3 RESULTS AND DISCUSSION

**FIGURE 2:**
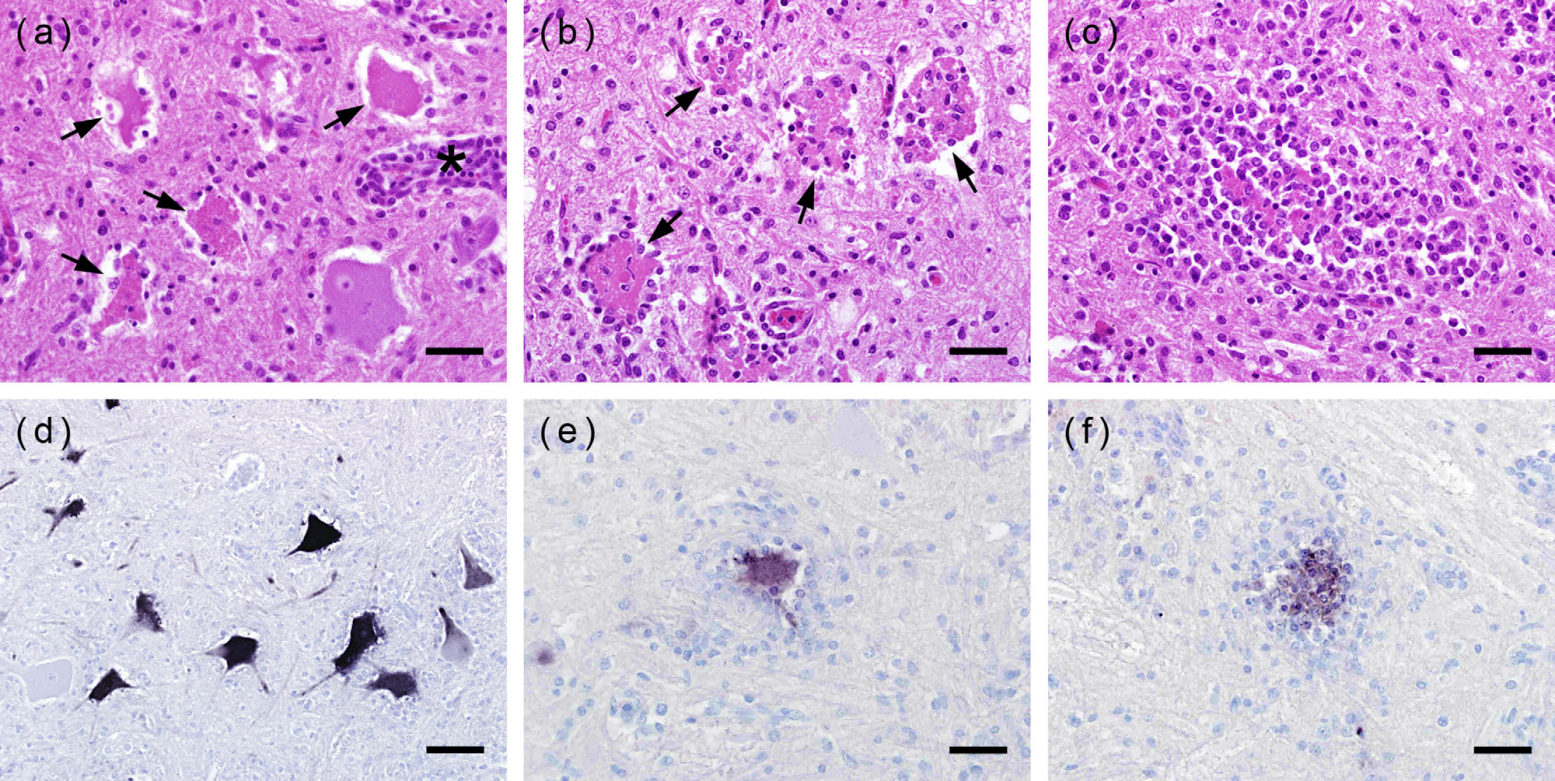
Histological lesions and localization of viral nucleic acid by in situ hybridization (ISH) in the spinal cord. (a) Lamb1: several necrotic neurons in the ventral horn with minimal glial reaction (arrows). A perivascular cuff (asterisk) indicates nonsuppurative myelitis. (b) Lamb 2: necrotic neurons (arrows) showing different stages of neuronophagia. (c) Lamb 1: a largely disintegrated and phagocytosed neuron surrounded by numerous glial cells. (d) Lamb 2: several morphologically largely intact neurons with minimal glial reaction show strong ISH signals. In the neurons with early (e) and advanced (f) stages of neuronophagia of lamb 1 the labelling is less prominent. (a–c): H.E. staining, (d–f): ISH; (a–c; e–f): bar = 40 μm; (d): bar = 80 μm

Necropsy of both animals did not reveal any obvious abnormalities. The histological lesions were identical in both animals and were confined to the central nervous system. The spinal cord was most severely affected. All levels showed a severe nonsuppurative myelitis, which was characterized by excessive necrosis (Figure 2a) or loss of neurons of both, the ventral and dorsal horns. In addition, there were numerous neuronophagic nodules (Figure 2b) as well as abundant gliosis (Figure 2c) and multiple lymphohistiocytic perivascular cuffs (Figure 2a). The lesions were particularly severe in the grey matter, but the perivascular cuffs extended into the white matter as well. The lesions in the brain were less pronounced and restricted to brainstem areas, such as thalamus, midbrain, medulla oblongata, and cerebellar roof nuclei. Here, perivascular cuffs and multifocal gliosis were the most prominent findings.

**FIGURE 3:**
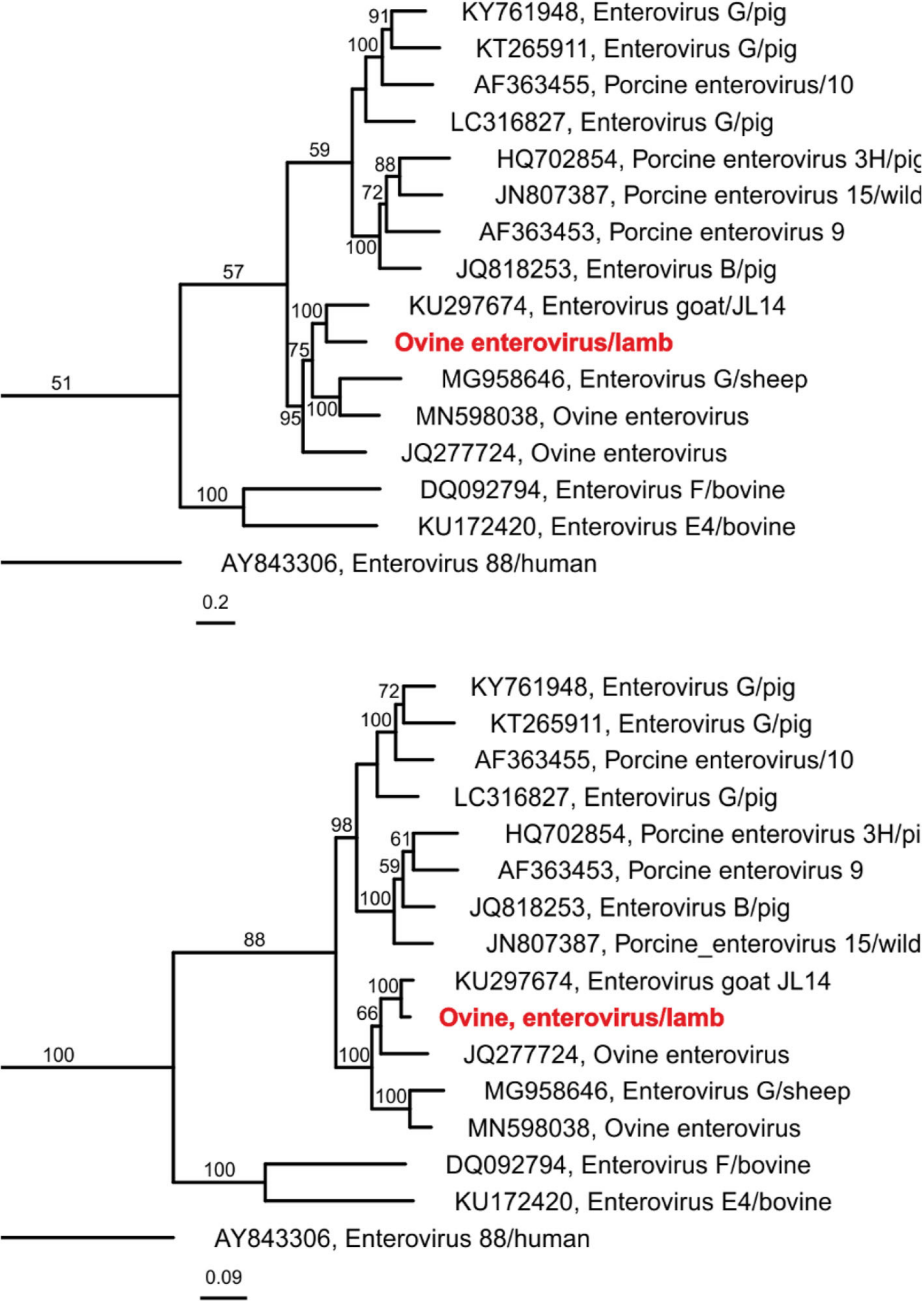
Phylogenetic analysis of full‐genome nucleotide sequences (upper) or polyprotein amino acid sequences (lower). For details, see text

Due to the striking histological similarities to a recently described picornavirus infection, native brain and spinal cord samples of lamb 1 were subjected to a specific RT‐qPCR assay (Forth et al., 2019), but the result was clearly negative. As the lesions were highly suggestive of a viral etiology, NGS‐based metagenomics was applied. RIEMS‐analysis of the initial dataset (2,018,160 reads total; 9821 reads removed for low quality; 2,008,257 reads classified) classified 31 reads as picornaviral sequences but revealed no other viruses nor significantly present bacteria (176 reads). To obtain a complete genome, additional data were generated (total reads final dataset 9.4 E+07 with 1.71 E+10 bases, after quality trimming 8.7 E+07 reads with 1.65 E+10 bases). With this approach, a nearly complete genome sequence of a hitherto unknown enterovirus could be assembled (INSDC databases, accession number: PRJEB47260). The obtained sequence has 7356 bases, a mean depth of 26.3 (±12.3), and a mean base quality of 63.8 (±2.4). It comprises one open reading frame that encodes one polyprotein with 2172 amino acids. Phylogenetic analysis revealed enteroviruses derived from goat and sheep as the closest relatives (Figure 3). These were an enterovirus found in goats with severe diarrhoea (M. Wang et al., 2017), an enterovirus found in the faeces of clinically healthy sheep (Boros et al., 2012), and two further ovine enteroviruses, the sequences of which are deposited in GenBank but no further information has been published.

By ISH with the specifically designed oligonucleotide probe using the novel sequence information, numerous motoneurons of the ventral horns of all spinal cord segments showed positive reactions. Labelling was most intense in morphologically intact neurons where it was located in the entire cell body (Figure 2d). Neurons with signs of necrosis or neuronal cell remnants within neuronophagic nodules sometimes also stained positively, but with less intensity (Figure 2e–f). Spinal cord areas with particularly strong glial reaction and inflammation occasionally did not show any signals of viral RNA. In the brain, there were only very scarce labelled neurons which were exclusively present in the brain stem. There was no specific signal in any other tissue.

Negative controls (spinal cord of lambs without neurological disease incubated with the enterovirus probe; spinal cords of the affected lambs incubated with an irrelevant PEDV probe) did not show any signals.

The detection of viral signals in the affected motoneurons confirms the presence of specific viral RNA in the respective lesions and indicates that the newly discovered enterovirus is most likely the causative agent of the associated clinical signs.

By RT‐qPCR, viral RNA was found in all examined spinal cord samples and in caudal brain areas, which corresponds well to the ISH results. In the majority of extraneural tissues, no viral RNA was identified; a low amount of viral RNA was detected in a liver, muscle, and intestinal sample of one animal, each (Table 1). In both animals, the concentration of viral nucleic acids in neural tissues was higher with a Cq difference of at least 5 between the highest load in neural compared to extraneural tissue.

**TABLE 1:** Results of reverse‐transcription quantitative PCR (RT‐qPCR) investigation of various samples of both affected animals

		Cq values			
		Lamb 1		Lamb 2	
	Sample	Enterovirus	ACTB	Enterovirus	ACTB
CNS	Frontal cortex	–	27.04	–	29.95
	Thalamus	–	26.12	–	37.65
	Hippocampus/midbrain	34.67	27.51	32.05	27.48
	Cerebellum	X	X	26.76	26.75
	Cervical spinal cord	29.14	29.26	33.18	36.74
	Thoracal spinal cord	X	X	34.53	35.88
	Lumbal spinal cord	29.67	28.03	36.69	39.81
Peripheral organs and nerves	Heart	–	24.29	–	23.75
	Lung	–	23.79	–	23.80
	Liver	34.42	25.61	–	24.01
	Spleen	–	22.53	–	23.15
	Kidney	–	23.91	–	24.45
	Pancreas	–	22.60	–	23.68
	Tongue	–	20.19	–	20.82
	Small intestine	–	22.85	37.27	23.89
	Inguinal lymph node	–	23.26	–	24.15
	Skeletal muscle	34.21	18.69	–	19.84
	Left sciatic nerve	–	27.17	–	28.46
	Right sciatic nerve	–	27.25	–	30.12

Abbreviation: ACTB, beta actin transcripts.

–: not detected.

X: not investigated.

In the last decade, particularly NGS‐based metagenomics contributed to significant progress in the elucidation of previously elusive causes of nonsuppurative encephalomyelitides in many animal species. The most outstanding achievements are the identification of astroviruses as causative agents in numerous species (Blomström et al., 2010; Matias Ferreyra et al., 2020; Pfaff et al., 2017; Selimovic‐Hamza et al., 2017), followed by sapeloviruses in pigs (Schock et al., 2014) and picornaviruses in sheep (Forth et al., 2019), as well as the probable association of bovine polyomavirus with encephalitis in cattle (Hierweger et al., 2020).

Especially enteroviruses are known to cause encephalomyelitis in several vertebrate species, and it is intriguing that there is a particular tropism to the spinal cord motor neurons. Brain lesions are comparatively less common. Two prominent disease entities caused by this group of viruses are poliomyelitis of humans (Melnick, 1996) and Teschen disease of pigs (Cantile & Sameh, 2016). The histological lesions of these entities are characterized by neuronal necrosis, neuronophagia, and glial nodules predominantly in the ventral horns of the spinal cord (Melnick, 1996; Pogranichniy et al., 2003). This seems to be a consistent feature of neurotropic enterovirus infections, independently of the affected vertebrate species and the relationships in the viral phylogeny. For human polioviruses, three wild type viruses have been recognized, two of which (wild type poliovirus 2 and 3) have been declared eradicated due to successful vaccination campaigns (Chard et al., 2020; Nathanson & Kew, 2010). In pigs, historically, porcine enteroviruses (serotypes 1 to 11) have been members of the genus Enterovirus in the Picornaviridae family. Recent reclassification has resulted in the establishment of two new genera, the Teschovirus genus (serotypes 1–7 and 11–13, now porcine teschovirus) and the Sapelovirus genus (serotype 8, now porcine sapelovirus A). Porcine enterovirus serotypes 9 and 10 (PEV‐9 and PEV‐10) remain in the Enterovirus genus (Alexandersen et al., 2019).

In accordance with the variation of human and porcine enteroviruses, it is very likely that a larger number of ovine enterovirus exist which are generally able to induce myelitis in lambs, probably with geographic restrictions. Within a short time span, two of them have been discovered, but it can be expected that more will emerge.

Novel enteroviruses may also be generated due to intraspecies and even interspecies recombination events. Boros et al. (2012) presented evidence that an enterovirus present in faecal samples from clinically healthy sheep was a natural recombinant of a porcine and bovine enterovirus.

Also, the circumstances of enterovirus pathogenicity (across species) are very similar. The viruses are widespread within their host species and the resulting herd immunity prevents the viruses from spreading from the intestine to the nervous system. This is the reason why enterovirus‐associated cases of (encephalo)myelitis are quite rare. Nervous system manifestation occurs in individuals with immunity gaps, either due to failure of colostrum uptake or at the time of weaning, when maternal immunity wanes and active immunity is not yet fully developed. Also, in the cases described here, a lack of colostrum uptake was evident in at least one lamb. Still, it cannot be ruled out that further lambs received insufficient colostrum since the lambs usually stay with their mothers for 5–10 days, and feed uptake is solely visually monitored and not further verified.

Taken together, this report describes a novel enterovirus which may be common in sheep and seems to be able to cause myelitis with severe motoneuron damage in colostrum‐deprived lambs. Thus, sheep farmers should be instructed that colostrum uptake has to be secured in order to provide adequate immunity to these potentially deleterious viral agents.

## CONFLICT OF INTEREST

The authors declare no conflict of interest.

## ETHICS STATEMENT

The authors confirm that the ethical policies of the journal, as noted on the journal's author guidelines page, have been adhered to. The authors confirm that no ethical approval was required as this work was carried out according to the rules pertaining to the clinical and diagnostic services of Vetmeduni Vienna.

## Data Availability

The data that support the findings of this study are available from the corresponding author upon reasonable request.
